# Supplementary material for: How early is too early? Challenges in ART initiation and engaging in HIV care under Treat All in Rwanda—A qualitative study
Source: PLoS One. 2021 May 13;16(5):e0251645. doi: 10.1371/journal.pone.0251645 (PMC8118273; doi:10.1371/journal.pone.0251645)
Supplement: S1 Checklist — (DOCX) [file pone.0251645.s001.docx]

**S1 Checklist: COREQ 32-item checklist^17^**

| Checklist item | Description | Reporting location |
| --- | --- | --- |
| Domain 1: Research team and reflexivity | | |
| 1. Interviewer | Which authors conducted the interview? | Methods, parag. 4 |
| 2. Credentials | What were the interviewer’s credentials? |  |
| 3. Occupation | What was their occupation at the time of the study? |  |
| 4. Gender | Was the interviewer male or female? |  |
| 5. Experience and training | What experience or training did the interviewer have? |  |
| Relationship with participants | | |
| 6. Relationship established | Was a relationship established prior to study commencement? | Methods, parag. 4 |
| 7. Participant knowledge of interviewer | What did participants know about the researcher? |  |
| 8. Interviewer characteristics | What characteristics were reported about the interviewer? |  |
| Domain 2: Study design | | |
| Theoretical framework | | |
| 9. Methodological orientation and Theory | What methodological orientation was stated to underpin the theory? | Methods, parag. 4 |
| Participant selection | | |
| 10. Sampling | How were participants selected? | Methods, parag. 3 |
| 11. Method of approach | How were participants approached? |  |
| 12. Sample size | How many participants were in the study? | Results, parag. 1 |
| 13. Non-participation | How many people refused to participate or dropped out? |  |
| Setting | | |
| 14. Setting of data collection | Where was the data collected? | Methods, parag. 4 |
| 15. Presence of non-participants | Was anyone else present besides the participants and researchers? |  |
| 16. Description of sample | What are important characteristics of the sample? | Results, parag. 1 |
| Data collection | | |
| 17. Interview guide | Were questions, prompts, guides provided by the authors? Was it pilot tested? | Results, Table 1; Methods, parag. 4 |
| 18. Repeat interviews | Were repeat interviews carried out? If yes, how many? | Methods, parag. 4 |
| 19. Audio/visual recording | Did the research use audio or visual recording to collect the data? |  |
| 20. Field notes | Were field notes made during and/or after the interview or focus group? |  |
| 21. Duration | What was the duration of the interviews or focus group? |  |
| 22. Data saturation | Was data saturation discussed? | Methods, parag. 5 |
| 23. Transcripts returned | Were transcripts returned to participants for comment and/or correction? |  |
| Domain 3: analysis and findings | | |
| Data analysis | | |
| 24. Number of data coders | How many data coders coded the data? | Methods, parag 5 |
| 25. Description of the coding tree | Did the authors provide a description of the coding tree? |  |
| 26. Derivation of themes | Were themes identified in advance or derived from the data? |  |
| 27. Software | What software, if applicable, was used to manage the data? |  |
| 28. Participant checking | Did participants provide feedback on the findings? |  |
| Reporting | | |
| 29. Quotations presented | Were participant quotations presented to illustrate the themes/findings? Was each quotation identified? | Results, all paragraphs |
| 30. Data and findings consistent | Was there consistency between the data presented and the findings? |  |
| 31. Clarity of major themes | Were major themes clearly presented in the findings? |  |
| 32. Clarity of minor themes | Is there a description of diverse cases or discussion of minor themes? |  |
