# Supplementary material for: How early is too early? Challenges in ART initiation and engaging in HIV care under Treat All in Rwanda—A qualitative study
Source: PLoS One. 2021 May 13;16(5):e0251645. doi: 10.1371/journal.pone.0251645 (PMC8118273; doi:10.1371/journal.pone.0251645)
Supplement: S1 Table — (DOCX) [file pone.0251645.s002.docx]

***Inzitizi n’abafasha mu gutanga no gukomeza gufata imiti igabanya ubukana bwa Virusi itera sida muri gahunda yo kuvura bose mu Rwanda (Treat all)***

***UMURONGO NGENDERWAHO W’IKIGANIRO KUBITABIRIYE –Ingingo 1.2***

*Murakoze kwemera nanone kwitabira ubu bushakashatsi.Turi gukora ubu bushakashatsi kugirango dusobanukirwe neza ku bintu bimwe na bimwe bifasha cyangwa bigora ababana n’ubwandu bw’agakoko gatera Sida mugutangira cg kuguma ku imiti igabanya virusi itera SIDA.Muri Rusange, Turifuza kumenya bihagije ubunararibonye bwanyu mukubona ubuvuzi n’imiti igabanya viusi itera Sida.Nubwo hari ibibazo byihariye ,intego y’iki kiganiro nukumva ubunanararibonye bwanyu.Mwibuke ko atari ngombwa gusubiza ikibazo icyaricyo cyose igihe wumva utisanzuye, kandi ushobora guhagarika ubwitabire igihe icyaricyo cyose .*

***Ibibazo by’ikiganiro***

- 1. *Igitsina (Gabo/ Gore)?*
  2. *Ufite imyaka ingahe?*
  3. *Ubu,waba ubona ubuvuzi bujyanye na virusi itera Sida?*
  4. *Niba ari yego :ku kihe kigo nderabuzima?Ni ryari?*

|  |  |
| --- | --- |
| **1.** | ***Wambwira uko byakugendekeye wisuzumisha ubwa mbere ugasanga ufite virusi itera Sida?***  *Baza Byimbitse:*   - *Mbwira uko byari bimeze mu byumweru bya mbere cyangwa amezi ya mbere* - *Mbwira uko wiyumvise umaze kwakira igisubizo* - *Ni iki utekereza cyakagombye kuba cyaroroheje igikorwa cyo kwisuzumisha?* - *Ni iki abakozi b’ikigo nderabuzima (Abaganga, abajyanama b’urungano, abajyanama b’ubuzima) bakora kugira ngo byoroherere abipimisha guhabwa igisubizo cy’uko bafite virusi itera SIDA?* - *Ni ubuhe bujyanama bwakagombye kuba bufasha cyane/ bufite akamaro kanini mu gihe umuntu ahabwa igisubizo cy’uko afite virusi itera SIDA?* - *Ni hehe wifuza guhererwa igisubizo cyawe nyuma yo kwisuzumisha ugasanga ufite virusi itera SIDA? (Urugero: Kwa muganga, mu rugo)* |
| **2.** | ***Wigeze ufata imiti igabanya ubukana bwa virusi itera sida?***  ***a) Niba ari oya: wambwira impamvu utayifata***   - *Baza Byimbitse:* - *Ni iki cyakubujije gutangira imiti?* - *Wambwira birambuye impamvu utari ku miti?*   ***b) Niba ari Yego:Wambwira ugutangira imiti kwawe bwambere:***  *Baza Byimbitse:*   - *Ushobora kumbwira uko byari bimeze ugitangira imiti mu minsi ya mbere ndetse n’ibyumweru bibanza?medications?* - *Wiyumvise ute mu mubiri ndetse n’amarangamutima ugitangira imiti?* |
| **3.** | ***Haba hari ibibazo cyangwa imbogamizi waba waragize byatumye gutangira imiti igabanya ubukana bwa virusi itera sida bikugora?***  *Baza byimbitse:*   - *Imbogamizi bwite/inzitizi* - *Imbogamizi zifitanye isano n’imiti* - *Imbogamizi zo ku kigo nderabuzima* - *Imbogamizi z’aho utuye* - ***Ibibazo bijyanye no kuvura bose*** |
| **4.** | ***Hari ibintu byakorohereje gutangira imiti?***  *Baza Byimbitse:*   - *ku mpamvu zawe bwite* - *ku mpamvu zifitanye isano n’imiti* - *ku mpamvu z’ikigo nderabuzima* - *ku mpamvu z’aho utuye* - ***ibibazo bishingiye ku kuvura bose*** |
| **5.** | ***Ni gute imiterere y’ikigo nderabuzima byagize uruhare mu gutangira imiti?***  *Baza Byimbitse:*  *- Kuboneka cyangwa kubura kw’abaganga*  *- Ingano y’igihe hagati yo kwisuzumisha no gutangira imiti*  *- ubujyanama mbere yo gutangira imiti*  *- Izindi serivisi zo kw’ivuriro zihari nizidahari* |
| **6.** | ***Hari ibindi bintu ikigo nderabuzima cg leta y’ u Rwanda byakora kugira ngo gutangira imiti byorohe?***  *Baza Byimbitse:*   - *Ni ibiki utekereza ku bijyanye no gutangira imiti ku munsi wasuzumiweho ugasanga ufite virusi itera SIDA?* - *? Ni igihe kingana iki utekereza ko gihagije hagati yo gusuzumwa ko ufite virusi itera SIDA no gutangira imiti?* - *Ni ubuhe bujyanama wifuza, wumva bwakoroshya igikorwa cyo gutangira imiti?* |

| **7.** | ***Ubu waba ufata imiti igabanya ubukana bwa virusi itera sida?***  ***a)*** *Niba ari Oya mbwira impamvu wayihagaritse(icyitonderwa :Niba atarigeze k’umuti jya ku kibazo gikurikira*  *Baza Byimbitse:*   - *Imbogamizi bwite/inzitizi* - *Imbogamizi zifitanye isano n’imiti* - *Imbogamizi zo ku kigo nderabuzima* - *Imbogamizi z’aho utuye* - ***Ibibazo bijyanye no kuvura bose***   ***b) Niba ari Yego:Mbwira imbogamizi izarizo zose waba warahuye nazo mu gukomeza byaburigihe gufata imiti igabanya virusi itera sida***  *Baza Byimbitse:*   - *Imbogamizi bwite/inzitizi* - *Imbogamizi zifitanye isano n’imiti* - *Imbogamizi zo ku kigo nderabuzima* - *Imbogamizi z’aho utuye* - ***Ibibazo bijyanye no kuvura bose*** |
| --- | --- |
| **8.** | ***Hari ibintu runaka byatuma kuguma ku miti bikorohera?***  *Baza Byimbitse:*   - *Impamvu bwite* - *Impamvu zifitanye isano n’imiti* - *Impamvu z’ikigo nderabuzima* - *Impamvu z’aho utuye* - ***Impamvu zijyanye no kuvura bose*** |
| **9.** | ***Hari ibindi bintu utekereza ikigo nderabuzima cyakora kugira ngo kuguma ku miti bikorohere? Ni ibihe?***  *Baza Byimbitse:*   - *Ni bande wifuza ko baguha imiti? (Abaforomo, ukora muri farumasi, umujyanama w’ubuzima, umujyanama w’urungano)* - *Ni kangahe wifuza ko wajya ujya gufata imiti kuri farumasi yo ku kigo nderabuzima (buri munsi, buri cyumweru, buri kwezi, buri mezi atatu)?* - *Ni iyihe miterere y’imiti wifuza gufata? (Ibinini, urushinge)* - *Ni kangahe wifuza kunywa imiti cyangwa guterwa urushinge? (buri munsi, buri cyumweru, buri kwezi, buri mezi atatu)?* |
| **10.** | **Mbwira ingorane wagize mu gukomeza kuza muri randevu z’ubuvuzi nkubana n’ubwandu bwa virusi itera sida**  *Baza Byimbitse:*   - *Imbogamizi bwite/inzitizi* - *Imbogamizi zifitanye isano n’imiti* - *Imbogamizi zo ku kigo nderabuzima* - *Imbogamizi z’aho utuye* - ***Ibibazo bijyanye no kuvura bose*** |
| **11.** | ***Hari ibintu runaka byakoroshya kuguma muri gahunda y’ubuvuzi ku kigo nderabuzima?Ni ibihe ?***  *Baza Byimbitse:*   - *Impamvu bwite* - *Impamvu zifitanye isano n’imiti* - *Impamvu zo ku kigo nderabuzima* - *Impamvu z’aho utuye* - ***Impamvu zijyanye no kuvura bose*** |
| **12.** | **Hari ibindi bintu utekereza ko ikigo nderabuzima cyakora mu rwego rwo koroshya iyubahirizwa rya randevu?Ni ibihe?**  *Baza Byimbitse:*   - *Ni hehe wifuza guhererwa ubuvuzi bwa virusi itera SIDA? (Ikigo nderabuzima, kuri posite do sante, ku mujynama w’ubuzima, mu rugo)* - *Ni ibiki utekereza ku bijyanye no gutandukanya serivisi za gahunda ya virusi itera SIDA n’izindi serivisi zo ku Kigo nderabuzima?* - *Ni ibiki utekereza ku bijyanye no guhuriza hamwe serivisi za gahunda ya virusi itera SIDA n’izindi serivisi zo ku kigo nderabuzima? Ni izihe serivisi za gahunda ya virusi itera SIDA wumva zahuzwa n’izindi servisi? Mu buhe buryo?* - *Ni ubuhe bujyanama wifuza ko wahabwa mu gihe uje muri gahunda y’ubuvuzi bwa virusi itera SIDA?* - *Ni iki utekereza ku ngano y’ubujynama uhabwa ku bijyanye na virusi itera SIDA? (Ni nyinshi cyane, nkeya cyane, iraringaniye/ irakwiye)* |
| **13.** | ***Leta y’urwanda yafashe umwanzuro wo gutangiza imiti igabanya ubukana bwa virusi itera sida umuntu wese basanganye virusi itera sida akayitangira vuba bishoboka uwo mwanzuro urawumva ute?*** |
| **14.** | ***Mu buvuzi uhabwa n’ibiki bibura cyangwa bidahagije nk’ubana na virus itera sida?*** |
| **15.** | ***Mu buvuzi uhabwa n’ibihe bintu ubona bitari ngombwa?*** |
