# Supplementary material for: How early is too early? Challenges in ART initiation and engaging in HIV care under Treat All in Rwanda—A qualitative study
Source: PLoS One. 2021 May 13;16(5):e0251645. doi: 10.1371/journal.pone.0251645 (PMC8118273; doi:10.1371/journal.pone.0251645)
Supplement: Attachment — Submitted filename: revision_response_to_reviewers.docx [file pone.0251645.s003.docx]

March 19^th^, 2021

Natasha McDonald, PhD

Editorial Office

PLOS ONE

Dear Dr. McDonald,

Thank you for your review of our manuscript entitled, “*How early is too early? Challenges in ART initiation and engaging in HIV care under Treat All in Rwanda – a qualitative study*.*”* We thank the reviewers for their comments, which were insightful and helpful. We have undertaken revisions in response to their suggestions and feel that these changes have substantially strengthened the manuscript. We have also addressed the editorial comments regarding style requirements, supporting information, and data availability. Below, we outline point-by-point responses to the comments by the reviewers, as well as the corresponding references in the revised manuscript.

| **Editor** |  |
| --- | --- |
| **1. Please ensure that your manuscript meets PLOS ONE's style requirements, including those for file naming.**  We have made changes to the formatting of the manuscript to meet PLOS ONE's requirements. |  |
| **2. If the questionnaire used in the study was used in a language other than English, please provide a copy as Supplemental Information to ensure that you have provided sufficient details that others could replicate the analyses.**  We have uploaded a Kinyarwanda version of the interview guide as Supplemental Information. |  |
| **3. Please include captions for your Supporting Information files at the end of your manuscript, and update any in-text citations to match accordingly.**  Supporting Information file captions were included after the References, as requested. |  |
| **4. We note that you have indicated that data from this study are available upon request. PLOS only allows data to be available upon request if there are legal or ethical restrictions on sharing data publicly.**  We have elaborated on data availability in the attached Cover Letter, as requested. |  |
| **5. We note that the grant information you provided in the ‘Funding Information’ and ‘Financial Disclosure’ sections do not match.**  We have noted in the cover letter that grant P30 AI124414 was not awarded directly to any of the study authors, rather, it supports the Einstein-Rockefeller-CUNY Center for AIDS Research. |  |
| **Reviewer #1** |  |
| **1. Inclusion criteria were: 1) ≥18 years; 2) living with HIV; 3) receiving/had received care from study health centers. For how long have they received care from the study centers? Duration on ART? 1 month, 2months, 12 months??**  We agree with the reviewer that additional information on the sample is relevant for context, and we have now included data on HIV diagnosis and time on ART, though these were not part of the inclusion criteria. Among the 37 participants included in the study, 7 were diagnosed prior to and 30 after Treat All implementation; median time from ART eligibility to ART initiation was 2 months, and median time on ART prior to the interview was 18 months (Page 10, Paragraph 1). |  |
| **2. What was the exclusion criteria?**  We thank the reviewer for identifying this omission. We note in the text that exclusion criteria were inability to speak Kinyarwanda, and inability to provide informed consent (Page 6, Paragraph 3). |  |
| **3. Participants were compensated 8,000 Rwandan francs ($8 USD). Compensation for what? Is compensation for their time and contribution? Was compensation given as an incentive to overcome barriers? The reason(s) for compensation should be explicit.**  Thank you for this comment. Participants were compensated for their time and for transportation to and from the health center for the research interview. We now state this in the manuscript (Page 6, Paragraph 3). |  |
| **4. The authors should briefly describe their relationship with participants: The relationship and extent of interaction between the researchers and their participants can influence participants’ responses. For transparency, the authors should identify and briefly state their assumptions and personal interests in the research topic.**  We now note in the methods that the study interviewers were not clinicians at the health centers and had no prior relationships with research participants, which we agree can provide better context for readers of the manuscript (Page 7, Paragraph 1). We also noted in the limitations that the interview setting may have resulted in social desirability bias (Page 19, Paragraph 2).  Additionally, we appreciate the suggestion to provide additional information regarding the authors’ background, interests and assumptions on the research topic, which we have done in the Methods (Page 5, Paragraph 3). |  |
|  |  |
| **5. Interview guides were iteratively refined to refined to clarify. (Please delete repeated words).**  We thank the reviewer for noticing this error, which has been corrected (Page 7, Paragraph 1). |  |
| **6. Was the interview guide pilot tested in this population? If No, why was it not pilot tested?**  Before starting data collection, we piloted the interview guide with Rwandan research staff not involved in the current study to ensure questions were comprehensible and interview length was appropriate, which we now note in the manuscript (Page 7, Paragraph 1). In line with best practices in qualitative research (Brod, et al, Qual Life Res 2009, throughout the data collection period, the guide was iteratively refined to clarify and further explore emerging themes relevant to implementation of Treat All. |  |
| **7. Data saturation was not considered in this study. Why was data saturation not considered during data collection?**  Based on study aims and available resources, we initially planned for a sample of approximately 30 participants, considered a reasonable estimate for achieving saturation in a sample (Baker, et al, National Centre for Research Methods Review Paper, 2012). While the logistics of conducting the study (i.e. need to conduct the analysis in English, duration of time needed for interview translation, and the geographic separation of the study team) limited our capacity to assess data saturation during the data collection process, regular discussions during the coding and analysis established that data saturation had been reached, as we now note in the text (Page 9, Paragraph 1). |  |
| **8. Were field notes made during and/or after the interviews/focus group? If yes, were they reflected in the results section?**  We thank the reviewer for this comment. Field notes were taken by one of the two research staff with the participants, while the other one conducted the interview. These notes were discussed during team meetings and incorporated into the analytic process, as is now noted in the methods (Page 7, Paragraph 1; Page 9, Paragraph 1). |  |
| **9. During the interviews, was anyone else present besides the participants and researchers? The authors should clarify.**  We have indicated in the text that only research participants and interviewers were present during the interviews (Page 7, Paragraph 1). |  |
| **10. Three investigators (CI, FU, JG) conducted a focus group with 8 study participants. How were these 8 participants selected?**  We have added text in the manuscript to describe how this group was purposefully selected to represent diverse viewpoints from the overall study sample in terms of gender, age, degree of clinical engagement (i.e. missed appointments); we note that all 8 participants who were invited to participate accepted (Page 9, Paragraph 1). |  |
| **11. Our findings also add to the emerging literature suggesting that emphasizing the social benefits of HIV treatment such as………….**  We have added to the text to provide examples of social benefits of HIV treatment such as an overall decreased risk of HIV acquisition in the population, a healthier population, etc. (Page 19, Paragraph 1). |  |
|  |  |
| **Reviewer #2** |  |
| **1. Consider the term “Treat All” vs. other terms (e.g., “test and treat”, “universal test and treat”, “rapid start”).**  Although both “Universal Test and Treat” and “Treat All” are utilized widely in the literature, we have chosen to use the latter term for several reasons. This is a term used by the World Health Organization to refer to policies it recommended in 2016 to provide ART for all people living with HIV, and to do so as soon as possible after diagnosis, and thus encompasses both the concepts of “universal treatment” as well as “rapid start.” Additionally, the term “Treat All” is preferentially used by the Rwandan Ministry of Health for clinical guidelines, research manuscripts and other publications. |  |
| **2. See the special issue in Global Public Health 16(2), which looks at the health system issue of “test and treat”. These papers may help to strengthen your introduction.**  We thank the reviewer for this suggestion; these articles were not available at the time of initial submission. We have reviewed this literature and incorporated several suggested citations into the introduction, highlighting similarities between Option B+ and Treat All with respect to potential impacts of policy implementation (Page 4, Paragraph 2). |  |
| **3. Were individuals eligible if they had initiated ART under PMTCT guidelines?**  As noted above (Reviewer 1, critique 1), eligibility was not limited to partcipants initiating ART under Treat All, and initial interviews included participants who had initiated ART under earlier eligibility guidelines. Because challenges initiating ART quickly after diagnosis emerged as a major theme, we thereafter focused recruitment towards participants who had first initiated ART after Treat All guideline implementation. Overall, 81% of the sample initiated ART after Treat All implementation, as noted in the manuscript (page 10, paragraph 1). |  |
| **4. Additional details are needed in describing the sample. Did all participants get diagnosed during the “Treat All” period? How many of the women had initiated ART under PMTCT? How long had participants been diagnosed? How much time elapsed for participants between HIV diagnosis and initiation of ART?**  Among all participants included in the study, median time to ART initiation from HIV diagnosis (for those initiating under Treat All) or ART eligibility (for those initiating prior to Treat All implementation) was 2 months; median time from ART initiation to the interview date was 18 months. This is now described in the text (page 10, paragraph 1). |  |
| **5. Define “missed appointment”.**  In the study health centers, if patients do not attend a scheduled clinical appointment, nurses call them to attend the subsequent day. If this attempt is unsuccessful, the appointment is marked as “missed” in the patient file. Health center nurses participating in recruitment efforts reviewed the patient files to target recruitment towards these patients. We have clarified this in the text (Page 6, Paragraph 3). |  |
| **6. Delete “All interviews were conducted in Kinyarwanda”. (previously stated in methods).**  We thank the reviewer for noting this redundancy, and have deleted the text accordingly (Page 10, Paragraph 1). |  |
| **7. Change “Traumatization by HIV diagnosis” to “Trauma of HIV diagnosis.”**  We agree with the reviewer that this terminology is clearer and have made the suggested change (Page 10, Paragraph 2). |  |
| **8. Lines 181-184 describe a participant who got diagnosed and then avoided care for 2 months before starting ART. As mentioned above, the “time elapsed” needs to be described for all participants.**  Thank you for this comment. As noted above, we have provided data on timing of HIV diagnosis and ART initiation, as well as duration on ART (Page 10, Paragraph 1). |  |
| **9. Lines 203-206 describes a participant who reported severe side effects. Is your impression that the reported side effects were truly a result of the medication, or psycho-somatic symptoms of the anxiety of a new diagnosis? I feel like that may deserve some exploration in the discussion, as this might be another potential drawback of a rapid start model, and an area for additional counseling.**  Side effects at ART initiation, particularly nausea, fatigue and dizziness, were commonly reported among participants in this study. During the study period, the most common first-line ART medications in Rwanda were tenofovir, lamivudine, efavirenz and abacavir, which commonly can cause these types of side effects. It is difficult – both as investigators and as clinicians – to disentangle to what degree these adverse effects may be driven by physiologic versus psycho-somatic processes. However, the overall impact on initiating ART of either mechanism, or both together, can be a negative one. We agree with the reviewer that counseling can emphasize the potential for adverse physiologic and psychosomatic effects of ART and provide patients with reassurance that these effects are typically short-lived, and comment briefly on this in the Discussion (Page 16, Paragraph 2). |  |
| **10. The word “contaminated” is often used in quotes. Consider the Kinyarwanda translation and whether the word “infected” would be a better translation.**  Thank you for this comment. The Kinyarwanda word participants used was *kwandura* which is best translated as “contaminated” or “made dirty.” Because this was the word used by participants, we feel that it is most appropriate to retain it in the quotes presented in the manuscript. We comment briefly on the use of this word as a reflection of the high levels of internalized stigma among participants in the Discussion (Page 17, Paragraph 2). |  |
| **11. The call for “trauma-informed care” related to the traumatic experience of an HIV diagnosis deserves more consideration. A trauma informed approach is an holistic approach that recognizes individuals’ broader life histories and how historical and on-going traumas impacts individuals’ health and engagement with the health system. In calling for trauma-informed care, this needs to go beyond the acknowledgement of the HIV diagnosis as a traumatic event. I would advise looking at the literature on diagnoses of other life-threatening illness (e.g., cancer) to see whether a trauma-informed care approach has been used specifically to process the traumatic event of a new diagnosis, and/or what other strategies exist to help individuals move beyond the initial shock and anxiety related to a new diagnosis.**  We very much appreciate this suggestion. We agree with the reviewer that trauma – beyond that of HIV diagnosis – is highly prevalent among PLWH, and that trauma and HIV intersect in fundamental ways that impact health and engagement in health care. This is particularly true in Rwanda, given the collective trauma of the 1994 conflict and genocide. We have expanded on the possibility of how trauma-informed care can be applied in the discussion (Page 17, Paragraph 1). |  |

Thank you for your continued interest and for considering this manuscript for publication in *PLOS ONE.* We very much appreciate the reviewers’ thoughtful critiques and feel that their comments have significantly improved this manuscript. We look forward to hearing back from you.

Sincerely,

Jonathan Ross MD, MS
